# Multimodal genomic testing in prenatal diagnosis: incremental diagnostic contribution of chromosomal microarray and exome sequencing, real-world NIPT confirmation rates and pregnancy outcomes in 120 consecutive amniocenteses from northeastern Türkiye — a retrospective cohort study

**DOI:** 10.1186/s12920-026-02491-7

**Published:** 2026-09-29

**Authors:** Çağrı Doğan, Selçuk Atalay, Hakan Timur

**Affiliations:** 1 Department of Medical Genetics, Ordu University Training and Research Hospital, Ordu, 52200 Türkiye; 2 Private Practice in Perinatology, Ordu, Türkiye; 3 Department of Obstetrics and Gynecology, Division of Perinatology, Ordu University, Ordu, Türkiye

**Keywords:** Prenatal diagnosis, Amniocentesis, Chromosomal microarray analysis, Copy number variants, Exome sequencing, Variant classification, Non-invasive prenatal testing, Confirmation rate, Genetic counselling, Türkiye

## Abstract

**Background:**

Most evidence on chromosomal microarray analysis (CMA) and exome sequencing (ES) in prenatal diagnosis comes from high-volume centres. We evaluated the incremental diagnostic contribution of additional testing modalities, non-invasive prenatal testing (NIPT) confirmation rates and pregnancy outcomes at a regional centre in Türkiye.

**Methods:**

Retrospective cohort of 120 consecutive amniocenteses (March 2022–June 2025). The pathway was retrospectively observed, not uniformly implemented: the intended first-tier panel (quantitative fluorescent PCR, G-banded chromosome analysis, CMA) was completed in 94 pregnancies (78.3%), CMA in 101 (84.2%); ES (*n* = 13) and targeted testing (*n* = 25) were selective. Findings were classified before analysis as fetal disease-causing or incidental/non-diagnostic. Cumulative yields use the whole cohort as denominator.

**Results:**

Abnormal findings occurred in 36 of 120 pregnancies (30.0%): 27 (22.5%) disease-causing and 9 (7.5%) incidental/non-diagnostic — 3 heterozygous pathogenic or likely pathogenic variants in autosomal recessive genes (2.5%) and 6 variants of uncertain significance (VUS; 5.0%). Disease-causing yield rose from 16.7% to 22.5%: seven of the 27 diagnoses were first identified by CMA, ES or targeted testing, a cohort-specific contribution, not a diagnostic yield, as these modalities were not applied uniformly. Abnormal ultrasonography was associated with an abnormal genetic finding (31/83 vs. 5/37; unadjusted OR 3.82, 95% CI 1.35–10.82); not significant for disease-causing findings alone. Within this referral stream, confirmation of 14 high-risk NIPT referrals was 6/6 for trisomies 21 and 18, 0/4 for sex chromosome and rare autosomal aneuploidies and 1/4 for copy-number variants; these should not be read as population-level positive predictive values. Outcome, sought only after an abnormal finding, was documented in 30 of 36; 22 were terminated (trisomy 18, 4/4; isolated VUS, 1/6).

**Conclusions:**

CMA, ES and targeted testing added diagnoses in a non-uniformly applied pathway (complete first-tier panel in 78.3%). The findings reinforce the importance of diagnostic confirmation of high-risk NIPT results before irreversible clinical decisions, particularly for less common targets, which were frequently not confirmed in this referral series. Termination frequencies varied across diagnostic categories; these descriptive differences should be considered in the context of the underlying fetal phenotype, diagnostic certainty, and parental preferences when counselling is provided.

**Supplementary Information:**

The online version contains supplementary material available at https://doi.org/10.1186/s12920-026-02491-7.

## Background

When a fetal structural anomaly or a high-risk screening result reaches a maternity service, the questions that follow are practical rather than technological: which test to request first, what a further tier adds, how much confidence a screening result deserves before it is discussed with the parents, and what can honestly be said about prognosis once results return. The answers determine what parents are told within a narrow decision window, often at an advanced gestational age.

The diagnostic tools available to answer these questions have changed rapidly. Amniocentesis remains the most widely used invasive procedure and has a well-established safety profile [1]. Conventional G-banded chromosome analysis resolves imbalances larger than approximately 5–10 Mb, whereas chromosomal microarray analysis (CMA) detects clinically significant copy number variants (CNVs) in about 6% of structurally anomalous fetuses with a normal karyotype [2] and is endorsed as a first-tier adjunct when fetal structural anomalies are present [3]. Quantitative fluorescent PCR (QF-PCR) reports the common aneuploidies within 24–48 h. For fetuses that remain undiagnosed, two landmark cohorts reported incremental exome sequencing (ES) yields of 8.5% and 10.3% beyond karyotype and CMA [4, 5], and a subsequent meta-analysis estimated a pooled incremental yield of 31% with wide variation by phenotype [6].

Non-invasive prenatal testing (NIPT) has meanwhile become the dominant first-line screen for common trisomies. Its positive predictive value (PPV), however, is strongly target-dependent: consistently high for trisomy 21 and substantially lower for trisomy 13, monosomy X and expanded panel targets [7–9]. Confirmatory invasive testing therefore remains essential, particularly for sex chromosome aneuploidies (SCAs) and microdeletion or microduplication syndromes [9, 10]. In routine obstetric practice the referral letter often states only that NIPT was “high risk”, and the counselling weight of that phrase differs by an order of magnitude depending on the target.

What happens after a diagnosis is equally variable. Reported termination rates reach 80–93% for trisomy 21 and 81–90% for trisomies 13 and 18, but only 33–58% for SCAs, with substantial variation between populations and legal frameworks [11–13]. Data from underserved regions of Türkiye, and from the eastern Black Sea in particular, are sparse; most national reports originate from high-volume centres in the west of the country [14, 15].

Much of this work is nevertheless done in regional maternity services that receive the referrals and deliver the counselling at moderate annual volume and without on-site access to every platform, and what a multimodal pathway delivers in that setting has rarely been reported.

We therefore analysed 120 consecutive amniocenteses performed over a 40-month period at a regional centre in northeastern Türkiye. Beyond regional diagnostic yield, we addressed three questions relevant to prenatal genomics outside high-volume centres: how much of the diagnostic information ultimately available to parents is generated by tests beyond conventional cytogenetics; what a high-risk NIPT referral is worth, by target category, within a real-world referral stream; and in which diagnostic categories irreversible decisions are taken. To avoid overstating fetal disease burden, findings were classified as either fetal disease-causing or incidental/non-diagnostic, using a dichotomy defined before the analysis was carried out; the second category is reported as two separate groups, heterozygous P/LP variants in autosomal recessive genes and variants of uncertain significance.

## Methods

### Design and setting

This was a single-centre retrospective cohort study of 120 consecutive amniocenteses performed at the Department of Medical Genetics, Ordu University Training and Research Hospital, a regional university hospital in northeastern Türkiye (eastern Black Sea region), between March 2022 and June 2025. Every pregnancy in which an amniocentesis was performed and a result issued from amniotic fluid was included, without exclusions. The pathway described below is a retrospectively observed clinical testing pathway rather than a prospectively applied algorithm: tests were requested according to indication, sample quality, turnaround requirements and the results of earlier tiers. All statements about tiers, increments and yields therefore describe what the service delivered in practice. Reporting follows the STROBE statement for cohort studies (Additional file 1).

### Participants and referral indications

Indications were classified into eight mutually exclusive categories: (1) abnormal fetal ultrasonography (fUSG) as the sole indication; (2) elevated maternal serum biochemical screening risk; (3) high-risk NIPT; (4) fUSG anomaly combined with one or more additional indications; (5) advanced maternal age (AMA, ≥ 35 years) without other indications; (6) previously affected child; (7) suspected fetal infection; and (8) other (patient request, familial spinal muscular atrophy carrier status, paternal chromosomal anomaly, aberrant right subclavian artery in a twin sibling). Where more than one indication was recorded, a high-risk NIPT result took precedence over all other indications, and an ultrasound anomaly combined with any further indication was assigned to category 4; this precedence rule is reproduced in the case-level dataset (Additional file 2, Table S1). The cohort comprised 118 singleton and two twin pregnancies: one referred because the co-twin had an aberrant right subclavian artery, and one dichorionic diamniotic pregnancy in which the co-twin had been selectively terminated at 14 weeks for a fetal anomaly and the surviving fetus was sampled at 16 weeks. A single sac was sampled in each, so every record in the case-level dataset corresponds to one procedure. Maternal age, gestational age at procedure, ultrasound findings, laboratory results and pregnancy outcome were extracted from the institutional electronic medical record.

### Observed clinical testing pathway

**Fig. 1:**
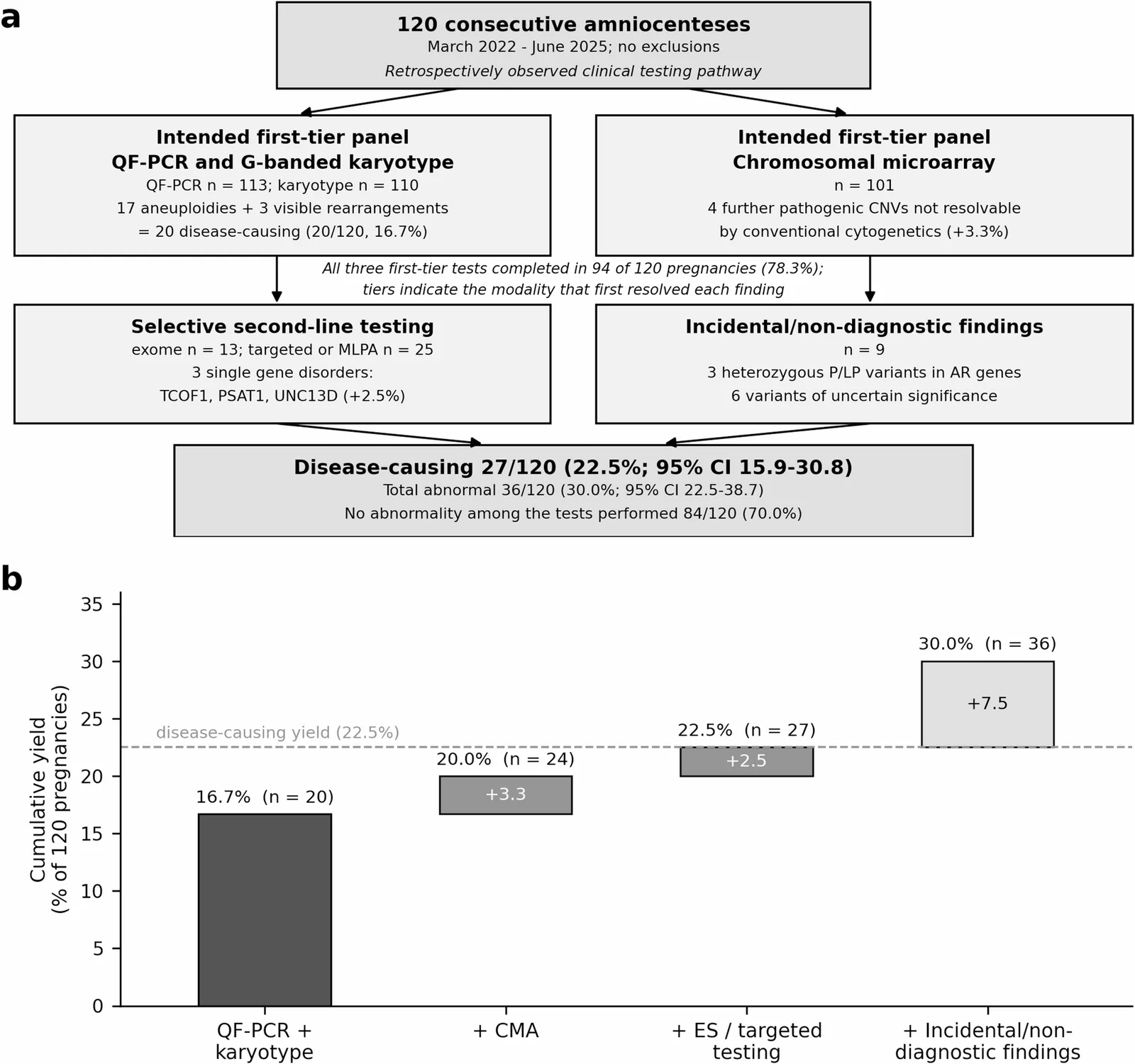
Testing flow and cumulative yield in 120 amniocenteses. **a** Flow of the retrospectively observed clinical testing pathway, showing the number of pregnancies tested with each modality and the findings first resolved by it. Tier labels indicate the modality that first resolved each finding; CMA formed part of the intended first-tier panel and is displayed separately for that reason. The incidental/non-diagnostic findings were identified by chromosomal microarray, exome sequencing and targeted testing; the position of that box reflects the layout of the panel rather than the modality of detection. **b** Cumulative disease-causing yield. QF-PCR and chromosome analysis, performed in 113 and 110 pregnancies respectively, identified 17 aneuploidies and three karyotypically visible unbalanced rearrangements (*n* = 20, 16.7%); CMA, performed in 101 pregnancies, identified four further pathogenic CNVs not resolvable by conventional cytogenetics (+ 3.3%); ES (*n* = 13) and targeted or MLPA analyses (*n* = 25) identified three single gene disorders (TCOF1, PSAT1, UNC13D; +2.5%), giving a disease-causing yield of 27/120 (22.5%; 95% CI 15.9–30.8). Cumulative and incremental yields use the whole cohort (*n* = 120) as the denominator and are specific to this cohort. Adding the nine incidental/non-diagnostic findings, which comprise three heterozygous P/LP variants in autosomal recessive genes (2.5%) and six variants of uncertain significance (5.0%), gives a total abnormal yield of 36/120 (30.0%; 95% CI 22.5–38.7)

Amniocentesis was performed under continuous ultrasound guidance and 15–20 mL of amniotic fluid was aspirated. The intended first-tier protocol comprised QF-PCR (AneuSure Kit, Genetek Biopharma, Türkiye; chromosomes 13, 18, 21, X and Y), G-banded chromosome analysis (400–550 bands, reported per ISCN 2020) and CMA on uncultured amniotic fluid DNA using the Affymetrix CytoScan Optima Array (Thermo Fisher Scientific, USA; approximately 315 000 probes; Chromosome Analysis Suite v3.1). Reporting thresholds were ≥ 200 kb for duplications and ≥ 50 kb for deletions in non-OMIM regions; CNVs intersecting OMIM-morbid genes were reported irrespective of size. CNVs were classified according to the ACMG/ClinGen technical standards for the interpretation and reporting of constitutional copy-number variants [16]. CMA formed part of the intended first-tier panel and was not used as a reflex second-tier test; in Fig. 1 and Table 3 it is nevertheless displayed as a separate tier because yield is attributed there to the modality that first resolved each finding, not to the order in which tests were requested. Genomic coordinates are reported on genome build GRCh38 throughout; intervals issued by the reporting laboratory on GRCh37 were converted to GRCh38 with the UCSC liftOver chain, and the original descriptions are otherwise unchanged.

The intended panel was applied pragmatically rather than uniformly. QF-PCR was performed in 113 pregnancies (94.2%), G-banded chromosome analysis in 110 (91.7%) and CMA in 101 (84.2%); all three first-tier tests were completed in 94 pregnancies (78.3%). Deviations arose in routine clinical practice, most often when a rapid result was requested, when culture was not established or when an earlier tier had already established a diagnosis; the reason for each deviation was not systematically recorded. The tests actually performed are reported for every pregnancy in Additional file 2 (Table S1). Two denominators are kept distinct throughout: the number of pregnancies in which a modality was performed, and the whole cohort (*n* = 120), which is used for the cumulative and incremental yields so that the tiers remain comparable. Incremental percentages are therefore cohort-wide proportions and not per-tier detection rates.

ES was performed selectively, on clinical grounds and after counselling of the parents, rather than in a predefined subgroup or in all undiagnosed pregnancies; any yield derived from it therefore applies only to the pregnancies selected for testing. ES was performed in 13 cases (10.8%) using GeneTopia Library Prep and Hybridization Capture Kits (approximately 51 Mb coding exome, targeting more than 99% of the regions in CCDS, RefSeq and GENCODE) on the GeneMind SURFSeq 5000 platform at a mean depth of 70–100×. Analysis covered coding exons and ± 10 bp of flanking intronic sequence; variants supported by 10 or fewer reads were not reported, and copy-number and structural variants were not called from ES data. Variants were filtered against gnomAD (v2.1.1), ExAC, 1000 Genomes and dbSNP at a minor allele frequency below 1%. Annotation and in silico assessment used Franklin (Genoox), VarSome, ClinVar, HGMD, DGV, DECIPHER, OMIM and Orphanet together with the predictor set implemented in the reporting pipeline, which combines missense and function predictors (SIFT, PROVEAN, MutationTaster, MutationAssessor, FATHMM, MetaSVM and MetaLR) with conservation metrics (GERP, phyloP, phastCons and SiPhy). Sequence variants were classified according to the ACMG/AMP standards and guidelines for the interpretation of sequence variants [17]. Trio analysis was performed when parental DNA was available; otherwise singleton ES was performed with parental Sanger segregation for pathogenic or likely pathogenic (P/LP) findings. Targeted single-gene sequencing or multiplex ligation-dependent probe amplification (MLPA) was performed in 25 cases (20.8%), including targeted TCOF1 sequencing in a pregnancy referred because of maternal Treacher Collins syndrome; CFTR analysis used Nextera XT library preparation and covered all coding exons and exon–intron boundaries (± 10 bp) of NM_000492.4, with variants supported by fewer than 20 reads not reported; MLPA used SALSA Probemix P091 (MRC Holland).

### Laboratory quality assurance

All diagnostic testing was carried out at a single external centre, licensed as a Genetic Diseases Evaluation Centre by the Turkish Ministry of Health and accredited as a medical testing laboratory to TS EN ISO 15189:2023 by the Turkish Accreditation Agency (TÜRKAK); every issued report identifies which of its analyses fall within the accredited scope. G-banded chromosome analysis was within that scope throughout the study period, whereas QF-PCR, chromosomal microarray and exome sequencing were not and rest on the laboratory’s own validation and routine quality control. All assays were in routine diagnostic use before the study period began, and none was developed for this analysis. Maternal cell contamination was assessed in amniotic fluid samples by short tandem repeat analysis of chromosomes 13, 18, 21, X and Y (AneuSure Kit, Genetek Biopharma, Türkiye) against a maternal blood sample taken at the time of the procedure. For G-banded analysis, two independent cultures were established per case and 20 metaphases examined at 400–550 bands, with additional cells counted when mosaicism was suspected and cultures repeated when these criteria were not met. The array was interpreted against PubMed, OMIM, DGV, DECIPHER and the laboratory’s in-house dataset, and every report states that balanced rearrangements, low-level mosaicism and regions not represented on the array are undetectable by the assay. Clinically significant sequence variants were confirmed by Sanger sequencing. Copy-number findings were confirmed selectively rather than universally: a second method was used when the result would alter clinical management, when the imbalance lay close to the reporting threshold or raised the possibility of mosaicism, or when it had first been seen on another platform (FISH for the 4p16.3 deletion, MLPA for the CFTR exon 23 duplication), while recurrent imbalances with an unambiguous array signature were issued on the array result alone. Because only the issued reports were available for this retrospective analysis, the DNA extraction platform and acceptance criteria, the QF-PCR allele ratio and peak height thresholds, the array quality metrics and the limit of mosaicism detection and the sequencing run acceptance criteria cannot be reported, and the prenatal chromosomal microarray and exome analyses are not covered by an external quality assessment scheme. Every result used here came from a final signed report; accreditation covers part of the test repertoire only and does not substitute for the analytical quality metrics of the individual assays, which is stated again among the limitations.

### NIPT and confirmation

NIPT had been performed at external commercial laboratories before referral, and the assay used was documented in the referral paperwork for part of the referral stream only. The predominant assay was the Illumina VeriSeq NIPT Solution v2, which analyses cell-free DNA by whole-genome sequencing and, in its genome-wide configuration, reports aneuploidy of all autosomes and, as an option, of the sex chromosomes, together with partial deletions and duplications of at least 7 Mb. The findings that generated the referrals analysed here (rare autosomal trisomies and segmental imbalances in addition to the common trisomies) are consistent with genome-wide rather than basic screening. Because platform metadata were not uniform across the referral stream, the confirmation rates reported here cannot be attributed to any single assay. For the 14 pregnancies referred after a high-risk NIPT result, the reported NIPT finding was compared with the amniocentesis result and the confirmation rate was calculated per target category, with Wilson score 95% confidence intervals. Because these referrals arose from routine clinical practice rather than a defined screening denominator, these values describe confirmation within this referral stream rather than assay performance, and no attempt was made to estimate sensitivity or specificity.

### Variant classification and clinical categorisation

To translate molecular results into categories usable in counselling, all findings were classified as (i) fetal disease-causing — an aneuploidy, a pathogenic or likely pathogenic CNV, or a variant or biallelic genotype expected to cause fetal disease in keeping with the inheritance pattern — or (ii) incidental/non-diagnostic. The second category is neutral and is reported as two separate groups that are never combined in a single count: heterozygous P/LP variants in autosomal recessive genes, which identify the fetus as a carrier, and variants of uncertain significance (VUS), whose relevance to the fetal phenotype could not be established. A VUS is not a carrier finding, and the two groups are given with their own numerators and denominators wherever the category is reported. This dichotomy is reported alongside conventional ACMG/AMP and ACMG/ClinGen classifications so that fetal disease yield is not inflated by findings that do not cause disease in the fetus. The term “no abnormality among the tests performed” means that no abnormality was identified among the genetic tests actually performed in that pregnancy. Because the intended panel was not completed in every case, the term does not imply that a complete genomic evaluation had been undertaken, and it is used with that meaning in the text, the tables, the figures and the case-level dataset (Additional file 2). Segmental imbalances of the X chromosome were classified as CNVs rather than as sex chromosome aneuploidies, since the mechanism, the counselling content and the recurrence risk differ; only whole-chromosome sex chromosome abnormalities are reported as SCAs. Parental segregation was performed by Sanger sequencing, ARMS-PCR or parental CMA as appropriate. All variants reported here were submitted to ClinVar under accession numbers SCV007657054–SCV007657068 (sequence variants) and SCV007657072–SCV007657082 (copy-number variants).

### Ultrasonographic assessment and outcome ascertainment

Fetal anomalies were grouped into seven categories (musculoskeletal or craniofacial, gastrointestinal markers, vascular and umbilical, increased nuchal translucency [NT] or nuchal fold or hydrops, central nervous system [CNS], cardiac, and choroid plexus cysts) by the reporting clinical geneticist, on the basis of the referring perinatologist’s report. Cases with anomalies in more than one organ system were assigned to all relevant categories, so the organ-system analysis is descriptive and its subgroups are neither mutually exclusive nor independent. Pregnancy outcome was ascertained retrospectively from the institutional electronic medical record and classified as live birth, termination of pregnancy (TOP) or outcome not ascertained. Follow-up was not attempted for the whole cohort: in routine practice at this department outcome is pursued only for pregnancies in which an abnormal finding has been reported, because these are the pregnancies recalled for post-test counselling. Outcome was therefore sought for the 36 pregnancies with an abnormal finding and not collected for the 84 with no abnormality among the tests performed. This is a structural feature of the service rather than random loss to follow-up, so the outcome analysis describes pregnancies with an abnormal finding only. No miscarriage, stillbirth or neonatal death was recorded among the pregnancies with a documented outcome; because follow-up was not attempted for the remainder, such events cannot be excluded there. All proportions are calculated within the documented group and no cohort-wide outcome rate is estimated. Post-amniocentesis complications were not systematically recorded during the study period and were not analysed, so the study does not permit estimation of a procedure-related complication rate.

### Statistical analysis

Continuous variables are summarised as mean ± standard deviation and median (range), and categorical variables as frequencies and percentages. Proportions were compared using Fisher exact tests, with odds ratios (OR) and 95% confidence intervals (CI) calculated by the Woolf logit method for 2 × 2 tables. All odds ratios are unadjusted and no multivariable modelling was performed. Wilson score 95% CIs were computed for binomial proportions and gestational age was compared using the Mann–Whitney U test. Analyses were performed in Python 3.11 with SciPy and statsmodels. No adjustment for multiple comparisons was applied and reported *p* values are nominal; a two-sided *p* < 0.05 was considered statistically significant. All subgroup analyses, including those by referral indication and by ultrasound category, are exploratory and descriptive, and no comparison between subgroups is offered as evidence of differential diagnostic performance. No formal sample size calculation was performed, as the study included all consecutive procedures over a fixed period.

### Use of large language models

The manuscript was designed and written by the authors. A large language model (Claude, Anthropic) was used solely to translate the text into English. It was not used to design the study, to generate, analyse or interpret data, or to draw conclusions. All clinical findings, variant classifications, statistical analyses and conclusions were produced and verified by the authors, who reviewed the translated text and take full responsibility for the integrity and accuracy of the content.

## Results

### Cohort and referral profile

Mean gestational age at amniocentesis was 18.1 ± 2.5 weeks (median 17, range 13–26), and 94 of 120 procedures (78.3%) were performed between 13 and 20 weeks. Four procedures (3.3%) were performed before 15 completed weeks, one at 13 + 6 weeks and three at 14 completed weeks. All four were referred with first-trimester ultrasound abnormalities: increased nuchal translucency in two, one with echogenic bowel; bilateral radial aplasia with additional limb anomalies, present at referral and preceding the procedure; and crown–rump length discordance with cystic hygroma and micrognathia in the pregnancy in which compound heterozygous PSAT1 variants were later identified. In each of these four pregnancies the anomaly had been documented in the first trimester and the diagnostic question was therefore already defined. Amniocentesis is ordinarily performed at or after 15 + 0 weeks, and sampling earlier than that was a deliberate departure from the recommended timing in these four pregnancies. Sampling went ahead at the parents’ request rather than being deferred: the first-trimester finding had generated considerable anxiety, and waiting until 15 + 0 weeks would have extended that period by about a week. The non-standard timing was discussed with the parents before each procedure, including the higher procedure-related risk reported for sampling before 15 + 0 weeks and the possibility that sampling at this gestation would fail to yield an informative result, and each procedure was carried out with their consent. All four yielded QF-PCR and a full karyotype, with CMA completed in three and ES in one. These figures describe sample adequacy alone: post-amniocentesis complications were not systematically recorded, so they carry no information about the safety of amniocentesis before 15 completed weeks. Mean maternal age was 31.5 ± 6.0 years (median 31, range 19–45) and 34 women (28.3%) were of advanced maternal age. The commonest indication was an isolated fUSG anomaly (50.0%), followed by biochemical screening risk (15.0%), high-risk NIPT (11.7%) and fUSG anomaly combined with an additional indication (11.7%) (Table 1). Taken together, ultrasound-driven indications accounted for 74 of 120 referrals (61.7%). Abnormal ultrasound findings were present in 83 cases (69.2%), most frequently vascular or umbilical anomalies (*n* = 28), gastrointestinal markers (*n* = 25), musculoskeletal or craniofacial anomalies (*n* = 24), choroid plexus cysts (*n* = 10), increased NT, nuchal fold or hydrops (*n* = 9), CNS anomalies (*n* = 8) and cardiac anomalies (*n* = 8).

**Table 1:** Distribution of amniocentesis indications (*n* = 120)

Indication	*n*	%
fUSG anomaly (sole indication)	60	50.0
Biochemical screening risk	18	15.0
High-risk NIPT	14	11.7
fUSG anomaly + additional indication	14	11.7
Other	5	4.2
Advanced maternal age (≥ 35 years)	3	2.5
Previously affected child	3	2.5
Fetal infection	3	2.5
Total	120	100.0

fUSG anomaly + additional indication includes fUSG combined with biochemical screening, advanced maternal age, previously affected child or maternal genetic disease. Where a high-risk NIPT result coexisted with an ultrasound anomaly the pregnancy was assigned to the NIPT category. Other comprises patient request (*n* = 2), familial SMA carrier status (*n* = 1), paternal chromosomal anomaly (*n* = 1) and ARSA in a twin sibling (*n* = 1)

*ARSA* aberrant right subclavian artery, *fUSG* fetal ultrasonography, *NIPT* non-invasive prenatal testing, SMA spinal muscular atrophy

### Overall yield and clinical categorisation

In 84 pregnancies (70.0%) no abnormality was identified among the genetic tests performed, and 36 (30.0%; 95% CI 22.5–38.7) had an abnormal finding (Table 2; Fig. 1). Twenty-seven (22.5%; 95% CI 15.9–30.8) carried disease-causing findings — 16 autosomal aneuploidies, 1 sex chromosome aneuploidy, 7 pathogenic CNVs and 3 single gene disorders — and 9 (7.5%) had incidental/non-diagnostic findings, comprising three heterozygous P/LP variants in autosomal recessive genes (3/120, 2.5%) and six variants of uncertain significance (6/120, 5.0%). The autosomal aneuploidies comprised trisomy 21 (*n* = 11), trisomy 18 (*n* = 4) and one fetus with trisomy 18 and a coexisting segmental X-chromosome imbalance; the single sex chromosome aneuploidy was a 47,XXX karyotype. The pathogenic or likely pathogenic CNVs were a 4p16.3 deletion (Wolf–Hirschhorn syndrome), a 1q41–q44 duplication, a 1q32.3–q41 deletion, a 16p11.2 deletion, a 2.0 Mb distal 17p13.3 deletion involving YWHAE and CRK but not PAFAH1B1, a 548 kb intragenic deletion of SOX6 at 11p15.2 and a recurrent 1.7 Mb Xp22.31 deletion encompassing STS, the region associated with X-linked ichthyosis.

**Table 2:** Amniocentesis results by diagnostic category and clinical classification (*n* = 120)

Diagnostic category	*n*	%
No abnormality among the tests performed	84	70.0
Disease-causing findings (subtotal)	27	22.5
Trisomy 21	11	9.2
Pathogenic CNV or microdeletion^a^	7	5.8
Trisomy 18	4	3.3
Single gene disease	3	2.5
Trisomy 18 with segmental X-chromosome imbalance	1	0.8
Sex chromosome aneuploidy (47,XXX)	1	0.8
Incidental/non-diagnostic findings (subtotal)	9	7.5
Heterozygous P/LP variants in autosomal recessive genes	3	2.5
Variants of uncertain significance	6	5.0
Total abnormal findings	36	30.0

Single gene diseases: TCOF1 (Treacher Collins syndrome, autosomal dominant, maternally inherited, detected by targeted sequencing); PSAT1 (autosomal recessive, compound heterozygous biparental); UNC13D (autosomal recessive, homozygous). Pathogenic or likely pathogenic CNVs: 4p16.3, 1q41–q44 gain, 1q32.3–q41, the distal 17p13.3 deletion involving YWHAE and CRK, 16p11.2, the intragenic SOX6 deletion at 11p15.2 and the recurrent Xp22.31 (STS) deletion. Incidental/non-diagnostic findings are reported as two separate groups. Carrier findings, that is heterozygous P/LP variants in autosomal recessive genes (3/120, 2.5%): MVK (with a co-occurring PIEZO2 VUS), GUCY2D and CFTR exon 23 duplication. Variants of uncertain significance (6/120, 5.0%): SLC34A1, co-occurring CFTR and PTPN11, SRPK3, TP63, FOXP1 c.1722G > A (synonymous, predicted splice-disrupting) and a de novo 166 kb Xp22.2 duplication involving MID1. “No abnormality among the tests performed” means that no abnormality was identified among the genetic tests actually performed in that pregnancy; because the complete intended first-tier panel was performed in 94 of 120 pregnancies, it does not imply that a complete genomic evaluation had been undertaken. Segmental X-chromosome imbalances are classified as CNVs, not as sex chromosome aneuploidies. Two further VUS (PIEZO2 and SCN5A) co-occur with other findings and are not tabulated separately to avoid double counting at case level (variant-level VUS total: *n* = 9 across 8 pregnancies). A fourth heterozygous P/LP variant in an autosomal recessive gene (SLC34A1 c.189del) similarly co-occurs with the homozygous UNC13D genotype and is not counted separately at case level

*CNV* copy number variant, *P/LP* pathogenic or likely pathogenic, *VUS* variant of uncertain significance

^a^The intragenic SOX6 deletion at 11p15.2 was issued as likely pathogenic; classification considered borderline and subject to revision as further evidence accumulates (see Discussion and Table S2)

Three single gene disorders (2.5%) were identified: Treacher Collins syndrome (TCOF1, autosomal dominant, maternally inherited from an affected mother and detected by targeted TCOF1 sequencing), phosphoserine aminotransferase deficiency (PSAT1, autosomal recessive, compound heterozygous biparental) and familial haemophagocytic lymphohistiocytosis type 3 (UNC13D, autosomal recessive, homozygous). Three heterozygous P/LP variants in autosomal recessive genes were classified as carrier findings within the incidental/non-diagnostic category (MVK, with a co-occurring PIEZO2 VUS; GUCY2D; and a de novo CFTR exon 23 duplication detected by MLPA). A fourth heterozygous pathogenic variant in an autosomal recessive gene, SLC34A1 c.189del, was identified alongside the homozygous UNC13D genotype and is therefore reported at variant level only (Additional file 2, Table S2). Six further pregnancies harboured a VUS as their only or principal finding: a heterozygous SLC34A1 missense variant; co-occurring CFTR and PTPN11 variants; SRPK3; TP63; FOXP1 c.1722G > A; and a de novo 166 kb Xp22.2 duplication. Two further VUS are reported at variant level only because they co-occur with another finding: an inherited SCN5A in-frame deletion alongside the 17p13.3 deletion, and the PIEZO2 variant alongside MVK. Variant-level detail is provided in Additional file 2 (Table S2).

Two findings illustrate why conservative classification matters in the prenatal setting. The FOXP1 c.1722G > A variant was confirmed de novo and predicted to disrupt splicing (SpliceAI Δ 0.92) but is synonymous at the protein level; supporting in silico evidence with de novo occurrence does not reach the threshold for likely pathogenic without functional confirmation, so it was reported as a VUS. The 166 kb Xp22.2 duplication (arr[GRCh38] Xp22.2(10,380,284–10,546,411)x2) also arose de novo, but its distal breakpoint falls within MID1, which carries a ClinGen haploinsufficiency score of 3 against a triplosensitivity score of 0, and the fetal phenotype did not correspond to Opitz G/BBB syndrome. It was therefore reported as a VUS rather than as a sex chromosome abnormality.

### What the pathway added beyond conventional cytogenetics

Yield accrued stepwise across the tiers (Table 3); because the modalities were not applied uniformly, these increments describe what this pathway delivered rather than a fully applied protocol. QF-PCR was performed in 113 pregnancies and G-banded chromosome analysis in 110, so at least 113 (94.2%) underwent one or both. Between them these modalities identified the 17 aneuploidies in the cohort and, as visible unbalanced rearrangements, three of the seven pathogenic CNVs — an add(6)(q27) subsequently resolved as a 1q41–q44 gain, a del(1)(q41q41) and a del(4)(p16) — giving 20 disease-causing diagnoses (20/120, 16.7%). CMA was performed in 101 pregnancies and contributed four further disease-causing diagnoses that conventional cytogenetics could not have resolved (the 16p11.2, 17p13.3, 11p15.2 and Xp22.31 deletions), raising cumulative disease-causing yield to 24/120 (20.0%); ES (*n* = 13) or targeted analyses (*n* = 25) identified three single gene disorders (3/120, 2.5%), giving a final disease-causing yield of 27/120 (22.5%). CMA and sequencing therefore added 5.8% points beyond conventional cytogenetics in this cohort: seven of the 27 disease-causing diagnoses (25.9%) were first identified by CMA, ES or targeted testing in this retrospectively observed clinical pathway and were not detectable by QF-PCR and conventional chromosome analysis alone; because these modalities were not applied uniformly, this figure represents the contribution observed in this cohort rather than an estimate of the diagnostic yield of these modalities. Because CMA was not performed in the remaining 19 pregnancies and sequencing was applied selectively, the increments of 3.3% and 2.5% are cohort-specific figures rather than universal detection rates. The 3.3%-point increase represents the contribution of CMA to the overall cohort and should not be interpreted as the diagnostic yield of CMA among the 101 pregnancies tested. In three further pregnancies conventional analysis would have identified an abnormality but CMA was required to define the imbalance and therefore the counselling content. Adding the nine incidental/non-diagnostic findings gives the total abnormal detection rate of 30.0%.

**Table 3:** Incremental diagnostic contribution by testing modality

Testing tier	*n* tested	Cumulative *n* (disease-causing)	Cumulative yield (%)	Incremental (%)
Chromosome analysis + QF-PCR	110 / 113	20	16.7	—
+ CMA	101	24	20.0	+ 3.3
+ ES or targeted testing	13 / 25	27	22.5	+ 2.5
+ Incidental/non-diagnostic findings	—	36	30.0	+ 7.5

The disease-causing column reflects fetal disease-causing findings only. The tiers describe a retrospectively observed clinical testing pathway and not a prospectively applied algorithm: not every pregnancy underwent every tier. Two denominators are used and are kept distinct. The second column gives the number of pregnancies in which each modality was actually performed (QF-PCR 113, G-banded chromosome analysis 110, CMA 101, ES 13, targeted testing or MLPA 25). The cumulative and incremental yields use the whole cohort (*n* = 120) as the denominator so that the tiers remain comparable; they are therefore cohort-wide proportions rather than per-tier detection rates, and they are specific to this cohort. For example, the incremental contribution of ES or targeted testing (+ 2.5%) represents three additional disease-causing diagnoses identified across the whole cohort of 120 pregnancies, and not a diagnostic yield of 2.5% among the pregnancies that actually underwent these tests. Tier 1 includes three unbalanced rearrangements visible on G-banded analysis (add(6)(q27), del(1)(q41q41), del(4)(p16)) whose precise gene content was defined only by CMA. Tier 2 counts only imbalances that conventional cytogenetics could not have resolved. The incremental contribution of CMA (+ 3.3%) likewise represents the contribution of the array to the whole cohort and is not the diagnostic yield of CMA among the 101 pregnancies in which it was performed. The final row adds the incidental/non-diagnostic findings, which comprise three heterozygous P/LP variants in autosomal recessive genes and six variants of uncertain significance, to give the total abnormal detection rate of 30.0%

*CMA* chromosomal microarray analysis, *ES* exome sequencing, *MLPA* multiplex ligation-dependent probe amplification, *QF-PCR* quantitative fluorescent PCR

### Yield by indication and by ultrasound findings

Yield varied markedly across indications (Table 4): fUSG anomaly with an additional indication 9 of 14 (64.3%), high-risk NIPT 7 of 14 (50.0%), advanced maternal age 1 of 3 (33.3%), previously affected child 1 of 3 (33.3%) and isolated fUSG anomaly 17 of 60 (28.3%). In an unadjusted comparison, an abnormal ultrasound was associated with an abnormal genetic result overall (31/83, 37.3% vs. 5/37, 13.5%; unadjusted OR 3.82, 95% CI 1.35–10.82, *p* = 0.009) (Table 5). When the outcome was restricted to disease-causing findings the association pointed in the same direction but did not reach significance (23/83, 27.7% vs. 4/37, 10.8%; unadjusted OR 3.16, 95% CI 1.01–9.92, *p* = 0.057). The four disease-causing findings in pregnancies with normal imaging were a recurrent Xp22.31 deletion, a 47,XXX karyotype and two cases of trisomy 21, referred for biochemical screening risk, advanced maternal age or a high-risk NIPT result. A normal ultrasound examination did not exclude a chromosomal diagnosis among pregnancies referred for invasive testing in this cohort. These estimates are not adjusted for maternal age, referral indication or gestational age and describe association rather than causation. The organ-system subgroups are small, ranging from 1 of 10 (10.0%) for choroid plexus cysts to 5 of 8 (62.5%) for cardiac anomalies; because pregnancies with multisystem anomalies contribute to several categories and no correction for multiple testing was applied, these figures are exploratory and descriptive and the categories should not be ranked on the basis of them. Yield was numerically higher in women of advanced maternal age (38.2% vs. 26.7%) but not significantly so (unadjusted OR 1.70, 95% CI 0.72–4.02, *p* = 0.270); 54.5% of trisomy 21 cases occurred in this group, meaning that nearly half occurred in women younger than 35. Gestational age at the procedure did not differ between pregnancies with an abnormal genetic finding and those with no abnormality among the tests performed (*p* = 0.247).

**Table 4:** Diagnostic yield by amniocentesis indication group

Indication group	*n*	Abnormal (*n*)	Yield (%)
fUSG anomaly + additional indication	14	9	64.3
High-risk NIPT	14	7	50.0
Advanced maternal age (≥ 35 years)	3	1	33.3
Previously affected child	3	1	33.3
fUSG anomaly (sole)	60	17	28.3
Biochemical screening risk	18	1	5.6
Fetal infection	3	0	0.0
Other	5	0	0.0

Yield refers to any abnormal genetic finding, including incidental/non-diagnostic findings. These subgroup yields are descriptive and exploratory

*fUSG* fetal ultrasonography, *NIPT* non-invasive prenatal testing

**Table 5:** Diagnostic yield by ultrasonographic finding category (any abnormal genetic finding)

Ultrasound finding category	Total (*n*)	Abnormal genetic (*n*)	Yield (%)
Vascular and umbilical	28	8	28.6
Gastrointestinal markers	25	6	24.0
Musculoskeletal or craniofacial	24	12	50.0
Choroid plexus cysts	10	1	10.0
Increased NT, NF or hydrops	9	4	44.4
Cardiac anomalies	8	5	62.5
Central nervous system	8	4	50.0
Any abnormal ultrasound	83	31	37.3
Normal ultrasound	37	5	13.5
Total cohort	120	36	30.0

Pregnancies with anomalies in more than one organ system were assigned to all relevant categories, so subcategory counts exceed the total and the subgroups are neither mutually exclusive nor independent; no correction for multiple testing was applied and the organ-system rows are exploratory and descriptive rather than ranked estimates. Rows are ordered by subgroup size, not by yield. Fisher exact test (any abnormal vs. normal ultrasound, all abnormal genetic findings): 31/83 (37.3%) vs. 5/37 (13.5%); unadjusted OR 3.82, 95% CI 1.35–10.82, *p* = 0.009. Restricted to disease-causing findings: 23/83 (27.7%) vs. 4/37 (10.8%); unadjusted OR 3.16, 95% CI 1.01–9.92, *p* = 0.057. Both odds ratios are unadjusted and describe association only

*CI* confidence interval, *NF* nuchal fold, *NT* nuchal translucency, *OR* odds ratio

### Sequencing findings and segregation

ES identified two disease-causing diagnoses (PSAT1 and UNC13D) among the 13 clinically selected pregnancies in which it was performed (2/13, 15.4%, 95% CI 4.3–42.2; 13 of 120, 10.8%). Because ES was not systematically applied, the observation is descriptive and cannot be used to estimate the diagnostic yield of ES in the overall cohort. The third single gene disorder, Treacher Collins syndrome, was diagnosed by targeted TCOF1 sequencing in a pregnancy referred because the mother was affected, and therefore falls outside the ES denominator. ES also detected heterozygous P/LP variants in autosomal recessive genes in two further pregnancies (MVK, GUCY2D) and a third such variant (SLC34A1) alongside the homozygous UNC13D genotype, so 4 of the 13 pregnancies carried a P/LP finding of any kind and 9 of 13 carried a P/LP finding or a VUS (the SLC34A1 variant, the co-occurring CFTR and PTPN11 variants, FOXP1, SRPK3 and the SCN5A variant found alongside the 17p13.3 deletion). Twenty-six of the 36 abnormal findings had arisen de novo, most of them aneuploidies; the remainder comprised the seven pathogenic or likely pathogenic CNVs, the CFTR exon 23 duplication, the Xp22.2 duplication and the FOXP1 variant. Inherited findings comprised two maternally inherited variants (TCOF1, GUCY2D), one paternally inherited variant (MVK), one compound heterozygous biparental genotype (PSAT1) and three homozygous biparental genotypes (UNC13D, SRPK3, TP63). Segregation remained unconfirmed in three pregnancies (the SLC34A1 variant of uncertain significance, the co-occurring CFTR and PTPN11 variants, and one trisomy 21).

### Confirmation of high-risk NIPT results

Among the 14 pregnancies referred after a high-risk NIPT result, the overall confirmation rate within this referral stream was 50.0% (7/14; 95% CI 26.8–73.2) (Table 6). All six high-risk NIPT referrals for trisomy 21 (*n* = 5) or trisomy 18 (*n* = 1) were confirmed in this referral series (6/6; 95% CI 61.0–100). Neither SCA call was confirmed (0/2, both reported as 47,XXY), and neither rare autosomal trisomy call was confirmed (0/2, one reported as trisomy 7 and 11 and one as trisomy 15); taken together, none of these four calls was confirmed (0/4; 95% CI 0–49.0). Of four NIPT-reported CNVs, only the 4p microdeletion was confirmed by CMA as Wolf–Hirschhorn syndrome (1/4; 95% CI 4.6–69.9); the 18q22 duplication, the Xq21.32–q23.3 deletion and the 2p24.3–p23.3 deletion were not confirmed. The confidence intervals are wide and overlap, so the gradient should be read as a direction rather than as a set of point estimates. All values in this section are confirmation rates within a referral stream and are neither positive predictive values nor measures of NIPT assay performance. A detection rate or sensitivity cannot be derived from them, because that would require a denominator containing all pregnancies with a confirmed abnormality, including those not identified by NIPT, which this referral-based design does not provide. Case-level NIPT–amniocentesis pairs are provided in Additional file 2 (Table S3).

**Table 6:** Confirmation of high-risk NIPT findings by amniocentesis (*n* = 14)

NIPT finding	*n*	Confirmed	Not confirmed	Confirmation rate (%)	95% CI
Trisomy 21	5	5	0	100.0	56.6–100
Trisomy 18	1	1	0	100.0	20.7–100
47,XXY	2	0	2	0.0	0–65.8
4p microdeletion	1	1	0	100.0	20.7–100
Trisomy 15	1	0	1	0.0	0–79.3
Trisomy 7/11	1	0	1	0.0	0–79.3
18q22 duplication	1	0	1	0.0	0–79.3
Xq21.32–q23.3 deletion	1	0	1	0.0	0–79.3
2p24.3–p23.3 deletion	1	0	1	0.0	0–79.3
Overall	14	7	7	50.0	26.8–73.2

Referrals arose from routine clinical practice rather than a defined screening denominator; values therefore describe confirmation rates within this referral stream and are neither positive predictive values in the epidemiological sense nor measures of NIPT assay performance. The predominant assay was the Illumina VeriSeq NIPT Solution v2 (whole-genome sequencing of cell-free DNA; genome-wide reporting of autosomal and sex chromosome aneuploidy and of partial imbalances of at least 7 Mb), but platform metadata were not uniform across the referral stream. Confidence intervals are Wilson score intervals. Grouped by target category: common autosomal trisomies 6/6 (100%; 95% CI 61.0–100), sex chromosome and rare autosomal aneuploidies 0/4 (0%; 95% CI 0–49.0), copy number variants 1/4 (25.0%; 95% CI 4.6–69.9)

*CI* confidence interval, *NIPT* non-invasive prenatal testing

### Pregnancy outcomes

**Fig. 2:**
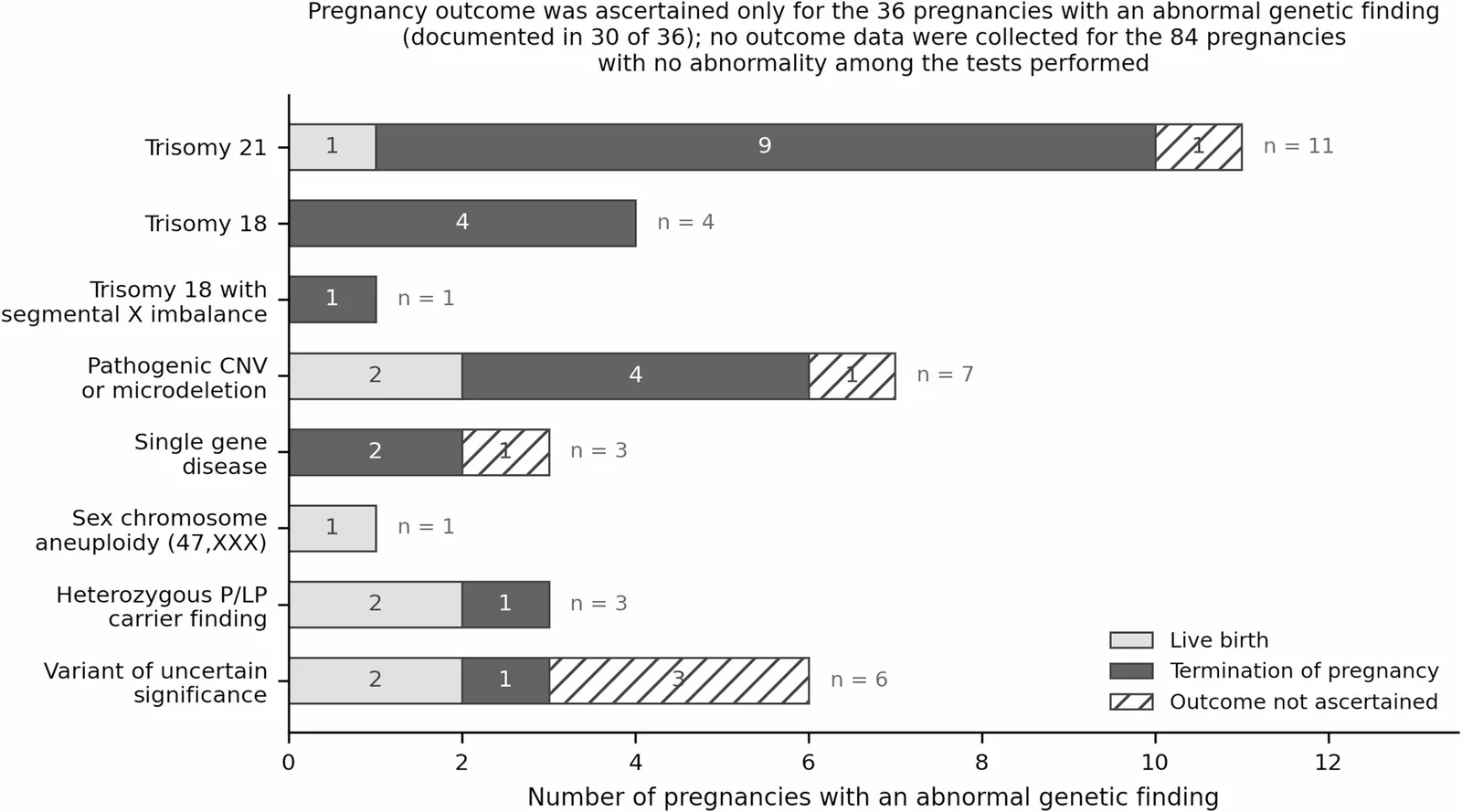
Pregnancy outcomes by diagnostic category among pregnancies with ascertained follow-up. Stacked horizontal bars show, for each diagnostic category, the number of live births, terminations and unascertained outcomes among the 36 pregnancies with an abnormal genetic finding. Outcome was sought only for these 36 pregnancies and was documented in 30 of them; no outcome data were collected for the 84 pregnancies with no abnormality among the tests performed, so the figure describes pregnancies with an abnormal finding only. Trisomy 18 (4/4) and the trisomy 18 with segmental X-chromosome imbalance (1/1) were terminated without exception, trisomy 21 in 9 of 11 pregnancies and pathogenic CNVs or microdeletions in 4 of 7. Findings without an expected fetal phenotype were rarely terminated: 1 of 3 heterozygous carrier findings and 1 of 6 pregnancies whose only finding was a variant of uncertain significance, while the single 47,XXX pregnancy resulted in live birth

Outcome was sought only for the 36 pregnancies with an abnormal genetic finding and was documented in 30 of them, which corresponds to 30 of the whole cohort of 120 (25.0%); outcome was not ascertained in the remaining 6 pregnancies with an abnormal finding, and no outcome data were collected for the 84 pregnancies with no abnormality among the tests performed (Table 7; Fig. 2). All proportions in this section therefore describe pregnancies with an abnormal finding and a documented outcome, and none of them can be extrapolated to the cohort as a whole or to pregnancies with no abnormality among the tests performed. Among these 30 pregnancies, 22 (73.3%) were terminated and 8 (26.7%) resulted in live birth. The proportion terminated varied by category, from 4 of 4 for trisomy 18 and 9 of 11 for trisomy 21 to 1 of 3 for heterozygous carrier findings and 1 of 6 where the only finding was a VUS, with the single 47,XXX pregnancy resulting in live birth (Table 7). Both mosaic trisomy 21 pregnancies were terminated. The single termination among the heterozygous carrier findings occurred in a pregnancy with fetal ascites, bilateral ventriculomegaly and angulation of the femoral head on ultrasonography. Among single gene disorders, the TCOF1 and PSAT1 pregnancies were terminated and outcome was not ascertained for the UNC13D pregnancy, nor for the Xp22.2 duplication or the FOXP1 pregnancy.

**Table 7:** Pregnancy outcome by diagnostic category among the 36 pregnancies with an abnormal genetic finding

Diagnostic category	Live birth	Termination	Outcome not ascertained	Total
Trisomy 21	1	9	1	11
Trisomy 18	0	4	0	4
Trisomy 18 with segmental X-chromosome imbalance	0	1	0	1
Sex chromosome aneuploidy (47,XXX)	1	0	0	1
Pathogenic CNV or microdeletion	2	4	1	7
Single gene disease	0	2	1	3
Heterozygous P/LP variant in autosomal recessive gene	2	1	0	3
Variant of uncertain significance	2	1	3	6
All abnormal findings	8	22	6	36
No abnormality among the tests performed (follow-up not attempted)	—	—	84	84
Total cohort	8	22	90	120

Pregnancy outcome was sought only for the 36 pregnancies with an abnormal genetic finding, because these are the pregnancies recalled for post-test counselling at this department, and was documented in 30 of them (30/120 of the cohort, 25.0%). No outcome data were collected for the 84 pregnancies with no abnormality among the tests performed, which are shown here as outcome not ascertained; the table therefore says nothing about that group and no proportion derived from it may be extrapolated to the cohort. Outcome was not ascertained for the Xp22.2 duplication involving MID1 or for the FOXP1 c.1722G > A pregnancy

*CNV* copy number variant, *P/LP* pathogenic or likely pathogenic, *VUS* variant of uncertain significance

Every termination in this series followed a genetic diagnosis or a reported abnormal finding; the reason recorded in the clinical notes was the genetic finding itself in 20 of the 22 terminations. In the remaining two, the recorded reason was the ultrasound phenotype: a lethal skeletal phenotype in the pregnancy in which compound heterozygous PSAT1 variants were identified, and bilateral ventriculomegaly in the pregnancy with the homozygous SRPK3 variant of uncertain significance. Nineteen of the 22 terminated pregnancies had abnormal ultrasound findings at referral; the remaining three had normal imaging and were terminated after a disease-causing diagnosis had been made (a recurrent Xp22.31 deletion, a mosaic trisomy 21 and a non-mosaic trisomy 21). Because outcome was not ascertained for any pregnancy with no abnormality among the tests performed, the study provides no information on how those pregnancies were managed.

## Discussion

### Principal findings

In this regional amniocentesis cohort the overall abnormal yield was 30.0%, comprising 22.5% disease-causing findings and 7.5% incidental/non-diagnostic findings (2.5% heterozygous P/LP variants in autosomal recessive genes and 5.0% variants of uncertain significance). Three observations extend beyond a descriptive account of regional yield, each specific to this cohort. Seven of the 27 disease-causing diagnoses were first identified by CMA, ES or targeted testing in this retrospectively observed clinical pathway and were not detectable by QF-PCR and conventional chromosome analysis alone; because these modalities were not applied uniformly, this figure represents the contribution observed in this cohort rather than an estimate of the diagnostic yield of these modalities. Confirmation of a high-risk NIPT result ranged from 6 of 6 for the common trisomies to 0 of 4 for sex chromosome and rare autosomal aneuploidies; these figures represent confirmation within a referral stream and should not be read as population-level positive predictive values. Among pregnancies with an abnormal finding and a documented outcome, termination frequencies varied across diagnostic categories, from 4 of 4 for trisomy 18 to 1 of 6 for pregnancies whose only finding was a variant of uncertain significance.

### What the additional modalities added in this service

The disease-causing yield of 22.5% sits within the 15–25% range reported in unselected amniocentesis cohorts [2, 18]. Cohort composition is the most likely explanation for its position in the upper half of that range: 69.2% of pregnancies had abnormal ultrasound findings at referral and isolated fUSG anomalies were the single largest indication. A cohort enriched for structurally abnormal fetuses will harbour more chromosomal and monogenic findings than one dominated by advanced maternal age or serum screening [4, 5].

The CMA-exclusive yield of 3.3% observed here is lower than the 6.0% incremental yield reported by Wapner et al. for structurally anomalous fetuses with a normal karyotype [2] and below the 5–9% range of subsequent studies [19, 20]. The difference is explained by how the increment is counted rather than by array performance: three of our seven pathogenic CNVs were already visible on G-banded analysis as unbalanced rearrangements, and studies that restrict the denominator to fetuses with a normal karyotype exclude exactly these cases. Counting only imbalances that conventional cytogenetics could not have resolved answers a different question — what is gained by adding the array rather than what is found with it — which is the question that arises when a service considers funding CMA. The resulting figure remains specific to this cohort and to the completeness with which each tier was applied, and it is a contribution to the whole cohort of 120 pregnancies rather than the diagnostic yield of CMA among the 101 pregnancies in which the array was performed. Even on this stricter definition, four pathogenic CNVs and three monogenic disorders — seven of the 27 disease-causing diagnoses — were first identified by CMA, ES or targeted testing in this retrospectively observed clinical pathway and were not detectable by QF-PCR and conventional chromosome analysis alone; because these modalities were not applied uniformly, this figure represents the contribution observed in this cohort rather than an estimate of the diagnostic yield of these modalities. In three further pregnancies the karyotype was abnormal but uninterpretable without CMA.

Published series are quoted here only as context: the pooled prenatal ES yield is 31% [6], and cohorts pre-selected on strict phenotypic criteria report 40–56% [21, 22]. ES was applied to 13 pregnancies chosen on clinical grounds, so the two diagnoses made in them are not comparable with those estimates and support no yield figure for this cohort. Targeted sequencing and MLPA, used in 25 pregnancies (20.8%), remain valuable for turnaround time, per-base coverage and cost [23, 24]; the Treacher Collins diagnosis, made by targeted TCOF1 sequencing in the fetus of an affected mother, is the clearest example, and the de novo CFTR exon 23 duplication exemplifies large intragenic rearrangements that sequencing-based approaches may miss [25].

These figures should be read against the completeness of the pathway itself. The full first-tier panel was delivered in 94 of 120 pregnancies (78.3%) and CMA in 101 (84.2%), so approximately one pregnancy in five did not receive the complete intended panel. What these data support is a service-level statement: a moderate-volume regional service delivered most components of the intended pathway to most pregnancies, and the later tiers changed what could be said to parents in those pregnancies in which they were applied.

### Interpreting a high-risk NIPT result in routine practice

The confirmation pattern observed here has immediate counselling consequences. All six high-risk NIPT referrals for trisomy 21 or trisomy 18 were confirmed in this referral series (6/6; 95% CI 61.0–100), none of the four SCA or rare autosomal trisomy referrals was confirmed, and one of the four CNV referrals was confirmed; the direction of this gradient matches the target-dependent pattern consolidated by Belabbes et al. [7]. Liu et al. reported PPVs of 86.1% for trisomy 21, 57.8% for trisomy 18, 20% for monosomy X and 68.8% for 47,XXY [8], and Li et al. found an overall PPV of 44.26% for SCAs across more than 22 000 pregnancies [9]. Our denominators are small and the CIs correspondingly wide, so no point estimate from this cohort should be quoted as a PPV; the direction, however, is consistent with much larger series. The phrase “high-risk NIPT” in a referral letter should not be treated as a single category. The confirmation of a 4p microdeletion as Wolf–Hirschhorn syndrome shows that expanded panels can be informative when ultrasound corroborates the finding [26, 27], while the three unconfirmed CNV calls reinforce that an isolated positive result should be confirmed by invasive testing before it shapes counselling [9, 10].

### What the outcome data can and cannot show

The outcome data must be read for what they are. Follow-up was pursued only for pregnancies in which an abnormal finding had been reported, so the outcome analysis describes that group and nothing else. Within it, termination frequencies varied substantially across diagnostic categories: they were highest for the autosomal trisomies and low for findings that carry no expected fetal phenotype (Table 7). No formal analysis of the determinants of these decisions was performed, and the differences should be read in the context of the underlying fetal phenotype, diagnostic certainty, parental preferences, gestational age at diagnosis and the legal and cultural setting. Three cases ran the other way and are worth stating explicitly. Two were driven by imaging rather than genotype: the pregnancy with a homozygous SRPK3 variant of uncertain significance, terminated for bilateral ventriculomegaly, and the pregnancy with compound heterozygous PSAT1 variants, terminated for a lethal skeletal phenotype. In the third, a recurrent Xp22.31 deletion predicting X-linked ichthyosis — a manageable condition — the pregnancy was nevertheless terminated, which shows that the category of the finding does not determine the decision and that parental values and the perceived certainty of the information carry independent weight. Because pregnancies with no abnormality among the tests performed were not followed, this study cannot say how such a result influenced management, and we make no claim about it.

### Separating fetal disease from incidental and non-diagnostic findings

Reporting disease-causing findings separately from incidental/non-diagnostic findings was a deliberate choice with counselling consequences. Heterozygous P/LP variants in autosomal recessive genes, and variants of uncertain significance, do not cause disease in the fetus, and conflating them with confirmed disease inflates the apparent burden of fetal disease by 7.5% points in this cohort. Reporting them transparently but separately preserves their reproductive value for the couple while protecting the accuracy of what is said about the current pregnancy.

Two cases show how finely balanced these judgements are, and in opposite directions. FOXP1 c.1722G > A could have been reported as likely pathogenic on an in silico splice prediction alone and could then have entered a termination discussion; reported as uncertain, it left postnatal transcript analysis open. The 11p15.2 deletion sits at the opposite boundary. It is a 548 kb loss, arr[GRCh38] 11p15.2(16,038,287–16,586,773), confirmed de novo and falling entirely within SOX6 (GRCh38 chr11:15,966,449–16,739,591), so it is an intragenic deletion of that gene. In favour of pathogenicity: SOX6 is associated with autosomal dominant Tolchin–Le Caignec syndrome (MIM 618971), the reported disease-causing alterations include intragenic microdeletions and truncating variants so haploinsufficiency is the established mechanism [28], and the deletion arose de novo and removes much of the coding region. Against it: the ClinGen dosage curation for SOX6 is open and the gene carries no established haploinsufficiency score, so the deletion cannot be scored against an established dosage-sensitive gene under the point-based system [16]; the fetal phenotype, a single umbilical artery with an echogenic intracardiac focus, is not specific for the gene; and intragenic deletions have not been reported often enough to establish penetrance. We report it within the disease-causing group because it was issued to the family as likely pathogenic, while stating that the classification is borderline and may be revised as further evidence accumulates; it is flagged as such in Table 2 and in Table S2. Were it reclassified as uncertain, the disease-causing yield would fall from 27 of 120 (22.5%) to 26 of 120 (21.7%) and the proportion of diagnoses first identified by CMA or sequencing in this non-uniformly applied pathway from 7 of 27 to 6 of 26, so the conclusions do not depend on this single classification. That pregnancy was terminated, which illustrates that conservative reporting is not costless in either direction. The Xp22.2 duplication involving MID1 is a third variation on the theme: a de novo, gene-disrupting duplication in a gene with strong haploinsufficiency but no triplosensitivity evidence, reported as uncertain.

### Classifying segmental X-chromosome imbalances

Three abnormal findings involved segmental imbalances of the X chromosome: the recurrent Xp22.31 deletion encompassing STS, the Xp22.2 duplication involving MID1, and an Xq23–q28 loss with an Xp22.33–p22.13 gain accompanying trisomy 18. Reporting practice often groups these with sex chromosome aneuploidies, but the counselling content differs: the Xp22.31 deletion in a male fetus predicts X-linked ichthyosis, which is manageable, informs neonatal care and carries a defined recurrence risk, whereas 47,XXX predicts a largely normal phenotype. Grouping them yields a category with no coherent counselling content and inflates the reported sex chromosome aneuploidy rate, so we report them separately.

### Timing of amniocentesis and access to care

Four procedures were performed before 15 + 0 weeks, the gestation from which the ISUOG practice guidelines recommend that amniocentesis be undertaken [29]. In each of them the fetal anomaly had been documented in the first trimester and the parents declined further delay after counselling. Because post-amniocentesis complications were not systematically recorded, these four procedures support no statement about the safety of sampling at that gestation. Almost a quarter of procedures (21.7%) were performed beyond 20 weeks, mostly reflecting delayed referral and anomalies first detected later in pregnancy [29, 30]. Late amniocentesis retains a high diagnostic yield with a low complication rate [30], and gestational age at the procedure did not differ between pregnancies with an abnormal genetic finding and those with no abnormality among the tests performed in this cohort (*p* = 0.247). Late testing is therefore defensible, but it compresses the time available for results and counselling, and the proportion of late procedures reflects access to timely fetal imaging rather than any laboratory characteristic.

### Recessive inheritance, screening indications and termination patterns

Most abnormal findings arose de novo, consistent with the predominance of aneuploidy, while the PSAT1 and UNC13D pregnancies underline the continued relevance of recessive inheritance in a population with elevated consanguinity [31]. The low yield among isolated biochemical screening referrals (1/18, 5.6%) fits a contemporary pathway in which many high-risk pregnancies undergo NIPT first [32], and the finding that nearly half of trisomy 21 cases occurred in women younger than 35 supports screening irrespective of maternal age [33, 34].

Termination frequencies differed between diagnostic categories. All four trisomy 18 pregnancies were terminated, as were 9 of 11 trisomy 21 pregnancies, close to the 67–85% reported in Western series [11] and the 79.6% reported by Herman et al. [13]. Pathogenic CNVs were terminated in 4 of 7. These denominators remain small, and cultural, religious and gestational-age considerations plausibly contribute to the differences between categories.

### Strengths and limitations

Strengths include consecutive enrolment without exclusions, standardised variant interpretation across the study period, explicit separation of fetal disease from incidental and non-diagnostic findings, with carrier findings and variants of uncertain significance reported apart from one another, separate reporting of segmental X-chromosome imbalances, conservative VUS classification, transparent reporting of the outcome data actually collected, release of the full case-level dataset, and STROBE-compliant reporting.

The limitations are substantial and constrain interpretation. The design is retrospective and single-centre with a modest sample size (*n* = 120). The testing pathway was observed retrospectively rather than implemented prospectively, and the intended first-tier panel was not completed in every pregnancy: all three first-tier tests were performed in 78.3% and CMA in 84.2%, so the tier-by-tier increments in Table 3 describe what the pathway delivered in practice rather than what a fully applied protocol would deliver, and the reasons for individual deviations were not systematically recorded. ES was performed in a clinically selected subgroup of 13 pregnancies (10.8%), so its yield applies to that subgroup only and this study cannot estimate the diagnostic yield of universal or phenotype-based ES. NIPT confirmation derives from 14 referrals arising from clinical practice rather than a defined screening denominator, so the estimates describe confirmation within this referral stream rather than assay performance. Outcome follow-up was pursued only for pregnancies with an abnormal finding and was documented in 30 of 120 (25.0%), with none collected for the 84 with no abnormality among the tests performed and 6 unascertained within the abnormal group; the outcome analysis therefore supports no inferential analysis and permits no statement about pregnancies with no abnormality among the tests performed. Consanguinity was not systematically captured; NIPT platform metadata were non-uniform; post-amniocentesis complications were not systematically recorded in this retrospective dataset, so the study does not permit estimation of the procedural complication rate and says nothing about the safety of the four procedures performed before 15 completed weeks; and ultrasound findings were grouped by a single reporter without duplicate coding, with the organ-system comparisons in Table 5 involving small, overlapping subgroups without adjustment for multiple testing. Laboratory quality assurance could be documented only from the issued reports, since testing was performed at an external centre: the DNA extraction platform and acceptance criteria, the QF-PCR allele ratio and peak height thresholds, the array quality metrics and the limit of mosaicism detection and the sequencing run acceptance criteria were not available for this analysis; the prenatal array and exome analyses are not covered by an external quality assessment scheme; and accreditation to TS EN ISO 15189:2023, which covers G-banded chromosome analysis but not QF-PCR, chromosomal microarray or exome sequencing, does not substitute for the analytical quality metrics of the individual assays. The classification of the 11p15.2 deletion and of FOXP1 c.1722G > A may change as functional and longitudinal data emerge.

## Conclusions

In this single-centre retrospective cohort of 120 consecutive amniocenteses, the implemented prenatal genetic testing pathway identified disease-causing genetic findings in 27 of 120 pregnancies (22.5%). Seven of these 27 diagnoses (25.9%) were first identified by CMA, ES or targeted testing in this retrospectively observed clinical pathway and were not detectable by QF-PCR and conventional chromosome analysis alone; because these modalities were not applied uniformly, this figure represents the contribution observed in this cohort rather than an estimate of the diagnostic yield of these modalities. CMA and selective sequencing therefore provided additional diagnostic information in this regional prenatal diagnostic service. However, because the intended testing pathway was not completed uniformly, ES was performed selectively in 13 pregnancies, the number of high-risk NIPT referrals was small (*n* = 14), and pregnancy outcome was sought only for the 36 pregnancies with an abnormal finding and documented in 30 of 120 (25.0%), these findings should not be interpreted as population-level estimates of assay performance or of pregnancy outcome. The findings reinforce the importance of diagnostic confirmation of high-risk NIPT results before irreversible clinical decisions, particularly for the less common targets, which were frequently not confirmed in this referral series; these observations represent confirmation within a referral stream and should not be read as population-level positive predictive values. Among pregnancies with an abnormal genetic finding and a documented outcome, termination frequencies varied across diagnostic categories. These descriptive differences should be interpreted in the context of the underlying fetal phenotype, diagnostic certainty, parental preferences and other clinical factors. Prospective multicentre studies with complete outcome ascertainment are required to determine the generalisability of these observations.

## Supplementary Information

Additional file 1. STROBE checklist. Completed STROBE checklist for cohort studies, with page references to the submitted manuscript.

Additional file 2. Supplementary Tables S1–S5. Table S1, de-identified case-level dataset for all 120 pregnancies (maternal age, gestational age, indication, ultrasound findings, tests actually performed, genetic result, diagnostic category and pregnancy outcome); Table S2, variant-level classification for the 36 abnormal findings; Table S3, case-level NIPT–amniocentesis confirmation pairs; Table S4, summary statistics computed directly from Table S1; Table S5, data dictionary.

## Data Availability

All variants reported in this study have been deposited in ClinVar (National Center for Biotechnology Information, https://www.ncbi.nlm.nih.gov/clinvar/). The 15 sequence variants identified by exome sequencing, targeted single-gene sequencing or MLPA are available under accession numbers SCV007657054–SCV007657068, and the 11 copy-number variants identified by chromosomal microarray are available under accession numbers SCV007657072–SCV007657082. The de-identified case-level dataset supporting the conclusions of this article, covering all 120 pregnancies, is provided as Additional file 2. Raw exome sequencing files (FASTQ and BAM) and raw microarray intensity files (CEL) are not publicly available because ethics approval for this retrospective study was granted with a waiver of individual informed consent that does not extend to public release of individual-level genomic data; these files are available from the corresponding author on reasonable request, subject to approval by the Ordu University Non-Interventional Clinical Research Ethics Committee and to a data transfer agreement.

## Abbreviations

ACMG: American College of Medical Genetics and Genomics
AMA: Advanced maternal age
AMP: Association for Molecular Pathology
ARSA: Aberrant right subclavian artery
CI: Confidence interval
ClinGen: Clinical Genome Resource
CMA: Chromosomal microarray analysis
CNS: Central nervous system
CNV: Copy number variant
ES: Exome sequencing
fUSG: Fetal ultrasonography
ISCN: International System for Human Cytogenomic Nomenclature
MLPA: Multiplex ligation-dependent probe amplification
NF: Nuchal fold
NIPT: Non-invasive prenatal testing
NT: Nuchal translucency
OR: Odds ratio
P/LP: Pathogenic or likely pathogenic
PPV: Positive predictive value
QF-PCR: Quantitative fluorescent polymerase chain reaction
SCA: Sex chromosome aneuploidy
SMA: Spinal muscular atrophy
STROBE: Strengthening the Reporting of Observational Studies in Epidemiology
TOP: Termination of pregnancy
VUS: Variant of uncertain significance
