# Supplementary material for: Multimodal genomic testing in prenatal diagnosis: incremental diagnostic contribution of chromosomal microarray and exome sequencing, real-world NIPT confirmation rates and pregnancy outcomes in 120 consecutive amniocenteses from northeastern Türkiye — a retrospective cohort study
Source: BMC Med Genomics. 2026 Sep 29;19:153. doi: 10.1186/s12920-026-02491-7 (PMC13640350; doi:10.1186/s12920-026-02491-7)
Supplement: Supplementary file 1 [file 12920_2026_2491_MOESM1_ESM.docx]

**STROBE Statement — Checklist of items that should be included in reports of cohort studies**

*Manuscript: “Multimodal genomic testing in prenatal diagnosis: incremental diagnostic contribution of chromosomal microarray and exome sequencing, real-world NIPT confirmation rates and pregnancy outcomes in 120 consecutive amniocenteses from northeastern Türkiye — a retrospective cohort study”*

*Submitted to BMC Medical Genomics — Corresponding author: Çağrı Doğan, MD — Fifth revision, September 2026*

| **Item No.** | **Recommendation** | **Reported on page(s)** |
| --- | --- | --- |
| **Title and abstract** | | |
| 1 | (a) Indicate the study’s design with a commonly used term in the title or the abstract  (b) Provide in the abstract an informative and balanced summary of what was done and what was found | Title, p. 1 (design stated in the title and in Methods of the Abstract); Abstract, pp. 1–2 |
| **Introduction** | | |
| 2 | Background/rationale: Explain the scientific background and rationale for the investigation being reported | Background, pp. 2–4 |
| 3 | Objectives: State specific objectives, including any prespecified hypotheses | Background, final paragraph, p. 4 |
| **Methods** | | |
| 4 | Study design: Present key elements of study design early in the paper | Methods, "Design and setting", p. 4 |
| 5 | Setting: Describe the setting, locations, and relevant dates, including periods of recruitment, exposure, follow-up, and data collection | Methods, "Design and setting", p. 4 (single centre; March 2022 – June 2025); "Ultrasonographic assessment and outcome ascertainment", p. 10 (follow-up) |
| 6 | (a) Cohort study—Give the eligibility criteria, and the sources and methods of selection of participants. Describe methods of follow-up  (b) Cohort study—For matched studies, give matching criteria and number of exposed and unexposed | Methods, "Design and setting" and "Participants and referral indications", pp. 4–5; follow-up in "Ultrasonographic assessment and outcome ascertainment", p. 10  N/A (not a matched study) |
| 7 | Variables: Clearly define all outcomes, exposures, predictors, potential confounders, and effect modifiers. Give diagnostic criteria, if applicable | Methods, "Observed clinical testing pathway" to "Ultrasonographic assessment and outcome ascertainment", pp. 5–10 |
| 8* | Data sources/measurement: For each variable of interest, give sources of data and details of methods of assessment (measurement). Describe comparability of assessment methods if there is more than one group | Methods, pp. 5–10; Additional file 2 (Table S1, case-level data; Table S5, data dictionary) |
| 9 | Bias: Describe any efforts to address potential sources of bias | Methods, "Observed clinical testing pathway", p. 6 (non-uniform application of the pathway); "NIPT and confirmation", p. 8 (referral-based denominator); "Ultrasonographic assessment and outcome ascertainment", p. 10 (outcome sought only for pregnancies with an abnormal finding); Discussion, "Strengths and limitations", pp. 24–25 |
| 10 | Study size: Explain how the study size was arrived at | Methods, "Design and setting", p. 4 (all consecutive procedures over a fixed period); "Statistical analysis", p. 10 (no formal sample size calculation) |
| 11 | Quantitative variables: Explain how quantitative variables were handled in the analyses. If applicable, describe which groupings were chosen and why | Methods, "Participants and referral indications", p. 5; "Ultrasonographic assessment and outcome ascertainment", p. 10; "Statistical analysis", p. 10 |
| 12 | (a) Describe all statistical methods, including those used to control for confounding  (b) Describe any methods used to examine subgroups and interactions  (c) Explain how missing data were addressed  (d) Cohort study—If applicable, explain how loss to follow-up was addressed  (e) Describe any sensitivity analyses | Methods, "Statistical analysis", p. 10  Methods, "Ultrasonographic assessment and outcome ascertainment", p. 10; Results, "Yield by indication and by ultrasound findings", p. 15  Methods, "Observed clinical testing pathway", p. 6; "Ultrasonographic assessment and outcome ascertainment", p. 10  Methods, "Ultrasonographic assessment and outcome ascertainment", p. 10; Discussion, "Strengths and limitations", pp. 24–25  Discussion, p. 22 (reclassification sensitivity statement for the 11p15.2 deletion) |
| **Results** | | |
| 13* | (a) Report numbers of individuals at each stage of study—eg numbers potentially eligible, examined for eligibility, confirmed eligible, included in the study, completing follow-up, and analysed  (b) Give reasons for non-participation at each stage  (c) Consider use of a flow diagram | Methods, "Design and setting", p. 4; Results, "Cohort and referral profile", p. 11; "Pregnancy outcomes", p. 17  Methods, "Observed clinical testing pathway", p. 6; Results, "Pregnancy outcomes", p. 17  Fig. 1 (legend p. 29) |
| 14* | (a) Give characteristics of study participants (eg demographic, clinical, social) and information on exposures and potential confounders  (b) Indicate number of participants with missing data for each variable of interest  (c) Cohort study—Summarise follow-up time (eg, average and total amount) | Results, "Cohort and referral profile", p. 11; Table 1 (p. 33)  Methods, "Observed clinical testing pathway", p. 6; Results, "Pregnancy outcomes", p. 17  Results, "Pregnancy outcomes", p. 17 (outcome sought for 36 pregnancies, documented in 30) |
| 15* | Outcome data: Cohort study—Report numbers of outcome events or summary measures over time | Results, pp. 12–18; Tables 2 and 7 (pp. 34, 38); Fig. 2 (legend p. 29) |
| 16 | (a) Give unadjusted estimates and, if applicable, confounder-adjusted estimates and their precision (eg, 95% confidence interval). Make clear which confounders were adjusted for and why they were included  (b) Report category boundaries when continuous variables were categorized  (c) If relevant, consider translating estimates of relative risk into absolute risk for a meaningful time period | Results, pp. 12–18; Tables 2–7 (pp. 34–38); all odds ratios reported as unadjusted, no confounder-adjusted estimates were computed  Methods, "Participants and referral indications", p. 5; "Statistical analysis", p. 10  N/A |
| 17 | Other analyses: Report other analyses done—eg analyses of subgroups and interactions, and sensitivity analyses | Results, "Yield by indication and by ultrasound findings", p. 15; "Sequencing findings and segregation", p. 16; Tables 4 and 5 (pp. 36–37) |
| **Discussion** | | |
| 18 | Key results: Summarise key results with reference to study objectives | Discussion, "Principal findings", p. 18; Conclusions, p. 25 |
| 19 | Limitations: Discuss limitations of the study, taking into account sources of potential bias or imprecision. Discuss both direction and magnitude of any potential bias | Discussion, "Strengths and limitations", pp. 24–25 |
| 20 | Interpretation: Give a cautious overall interpretation of results considering objectives, limitations, multiplicity of analyses, results from similar studies, and other relevant evidence | Discussion, pp. 18–25 |
| 21 | Generalisability: Discuss the generalisability (external validity) of the study results | Discussion, "What the additional modalities added in this service", pp. 19–20; "Strengths and limitations", pp. 24–25; Conclusions, p. 25 |
| **Other information** | | |
| 22 | Funding: Give the source of funding and the role of the funders for the present study and, if applicable, for the original study on which the present article is based | Declarations, "Funding", p. 28 |

**Give information separately for exposed and unexposed groups (cohort studies), cases and controls (case-control studies), or comparison groups (cross-sectional studies). In this single-cohort prenatal diagnostic study, no exposed/unexposed comparison applies; data are reported for the whole cohort and for prespecified clinical subgroups (e.g., abnormal vs. normal ultrasonography).*

*Note: An Explanation and Elaboration article discusses each checklist item and gives methodological background and published examples of transparent reporting. The STROBE checklist is best used in conjunction with this article (freely available on the Web sites of PLoS Medicine at http://www.plosmedicine.org/, Annals of Internal Medicine at http://www.annals.org/, and Epidemiology at http://www.epidem.com/). Information on the STROBE Initiative is available at www.strobe-statement.org.*

*Page numbers refer to the revised manuscript file (Amniocentesis_BMCMedGenomics_Manuscript_R5.docx). Because pagination can shift between word-processor versions, the section in which each item is reported is given alongside the page number.*
